# Performance of bedside tools for predicting infection-related mortality and administrative data for sepsis surveillance: An observational cohort study

**DOI:** 10.1371/journal.pone.0280228

**Published:** 2023-03-02

**Authors:** Meghan Bateson, Charis A. Marwick, Harry J. Staines, Andrea Patton, Elaine Stewart, Kevin D. Rooney

**Affiliations:** 1 ihub, Healthcare Improvement Scotland, Glasgow, United Kingdom; 2 Population Health & Genomics Division, School of Medicine, University of Dundee, Dundee, United Kingdom; 3 Healthcare Biometrics, Sigma Statistical Services, Balmullo, United Kingdom; 4 Usher Institute, University of Edinburgh, Edinburgh, United Kingdom; 5 School of Health and Life Sciences, University of the West of Scotland, Lanarkshire, United Kingdom; 6 Department of Anaesthetics and Intensive Care Medicine, Royal Alexandra Hospital, Paisley, United Kingdom; Heidelberg University Hospital, GERMANY

## Abstract

**Background:**

Measuring sepsis incidence and associated mortality at scale using administrative data is hampered by variation in diagnostic coding. This study aimed first to compare how well bedside severity scores predict 30-day mortality in hospitalised patients with infection, then to assess the ability of combinations of administrative data items to identify patients with sepsis.

**Methods:**

This retrospective case note review examined 958 adult hospital admissions between October 2015 and March 2016. Admissions with blood culture sampling were matched 1:1 to admissions without a blood culture. Case note review data were linked to discharge coding and mortality. For patients with infection the performance characteristics of Sequential Organ Failure Assessment (SOFA), National Early Warning System (NEWS), quick SOFA (qSOFA), and Systemic Inflammatory Response Syndrome (SIRS) were calculated for predicting 30-day mortality. Next, the performance characteristics of administrative data (blood cultures and discharge codes) for identifying patients with sepsis, defined as SOFA ≥2 because of infection, were calculated.

**Results:**

Infection was documented in 630 (65.8%) admissions and 347 (55.1%) patients with infection had sepsis. NEWS (Area Under the Receiver Operating Characteristic, AUROC 0.78 95%CI 0.72–0.83) and SOFA (AUROC 0.77, 95%CI 0.72–0.83), performed similarly well for prediction of 30-day mortality. Having an infection and/or sepsis International Classification of Diseases, Tenth Revision (ICD-10) code (AUROC 0.68, 95%CI 0.64–0.71) performed as well in identifying patients with sepsis as having at least one of: an infection code; sepsis code, or; blood culture (AUROC 0.68, 95%CI 0.65–0.71), Sepsis codes (AUROC 0.53, 95%CI 0.49–0.57) and positive blood cultures (AUROC 0.52, 95%CI 0.49–0.56) performed least well.

**Conclusions:**

SOFA and NEWS best predicted 30-day mortality in patients with infection. Sepsis ICD-10 codes lack sensitivity. For health systems without suitable electronic health records, blood culture sampling has potential utility as a clinical component of a proxy marker for sepsis surveillance.

## Introduction

Sepsis, redefined in 2016 as “life-threatening organ dysfunction caused by a dysregulated host response to infection” [1], is globally estimated to cause between five and 11 million deaths per year [2,3]. Survivors of sepsis are at increased risk of hospital readmission and long-term functional sequelae [4,5]. Sepsis related morbidity, mortality and consequent financial burden [6,7] has ignited an international focus on improving the prevention, recognition, and management of sepsis [8]. Evaluating the impact of changes intended to improve practice requires accurate surveillance of sepsis incidence and outcomes which is stable over time.

Sepsis identification and surveillance have been complicated by changes in definitions over time and the number of clinical tools used to screen for sepsis. In 2016, the Sepsis-3 [1] consensus guidelines set out diagnostic criteria for sepsis as organ dysfunction, defined as an acute increase in total Sequential [Sepsis-Related] Organ Failure Assessment (SOFA) [9] score of at least two, as a consequence of infection. Using the Sepsis-3 definition in emergency departments and general wards is challenging since SOFA is not routinely used outside of critical care. Furthermore, SOFA is calculated only once per 24 hours which limits its ability to detect sepsis early, crucial to the improvement of patient outcomes [10].

In recognition of the need for a bedside tool to identify patients with infection at risk of poor outcomes the Sepsis-3 guidelines recommended the use of qSOFA (quick SOFA) outside critical care [1]. Recently however, due to evidence that qSOFA lacks sensitivity the Surviving Sepsis Campaign guidelines have cautioned against its use as a standalone sepsis screening tool [11]. Other severity scores commonly in use in the United Kingdom, and internationally, include the National Early Warning Score (NEWS & NEWS2) [12,13] and Systemic Inflammatory Response Syndrome (SIRS) criteria [14]. However, the scores differ in their ability to identify patients with infection at high risk of death [15,16]. There is a need to establish how these scores compare to SOFA, as the Sepsis-3 [1] gold standard, to inform the development of a clinically relevant measure for sepsis surveillance.

The development of sepsis surveillance measures using clinical rather than administrative data is desirable because of concerns around inconsistency in the application of administrative codes to patients with sepsis [17]. While electronic health records have the potential to enable sepsis surveillance based on clinical criteria, national use of electronic health records is not yet widespread even in high income countries making purely clinically based measures impractical for many settings. Meantime, there is a need for an objective and reliable method of identifying the population with sepsis at scale to monitor outcomes. The most commonly used administrative data for tracking epidemiological trends are the International Classification of Diseases, Tenth Revision (ICD-10) codes [18]. The use of ICD-10 codes to track sepsis is controversial due to changes in definition, trends in coding practice and the range of potentially appropriate codes [19]. Following the implementation of National Health Service (NHS) Digital Coding Guidance NHS England reported a doubling in the use of sepsis codes (A40, streptococcal sepsis; A41, other sepsis) between 2016/17 [20] and 2017/2018 [21]. Sudden changes in coding practice are multifactorial but introduce problems in using ICD-10 codes as an isolated method for sepsis surveillance, risking misinterpretation of such changes as changes in sepsis incidence [22,23].

Including clinical data such as blood cultures in a surveillance measure for sepsis may offer some mitigation for the caveats of coding, but it has potential limitations. There will be a proportion of patients who do not have blood cultures obtained despite meeting clinical criteria to do so, leading to data which are missing not at random. Despite this there remains a need for a clinically relevant method to identify patients with sepsis which can be applied at scale, ideally from routinely collected administrative data [22–24].

Our aims were:

1. To calculate the performance characteristics of clinical severity scores (SOFA, qSOFA, NEWS, SIRS) in predicting the 30-day mortality of people with infection.
2. To calculate the performance characteristics of administrative data (ICD-10 codes and blood culture sampling) in identifying people with sepsis, defined as SOFA ≥2 as a consequence of infection.

## Methods

The data were generated as part of routine NHS care. The Caldicott Guardian for NHS Greater Glasgow & Clyde, who is responsible for protecting confidentiality of people’s health and care information and making sure it is used appropriately, gave permission for access to the data. The data were anonymised for analysis at the earliest opportunity. The West of Scotland Research Ethics Committee (ref: 17/WS/0044) approved the study without the need for informed consent.

### Patient population

This was a single-centre retrospective case note review of adult patients admitted to a large district general hospital in Greater Glasgow & Clyde, in the west of Scotland, United Kingdom. We identified all adult (≥16 years) admissions between 1^st^ October 2015 and 31^st^ March 2016 from the hospital administration system and linked them to blood culture data from the hospital microbiology system. Patients admitted to maternity units were excluded due to differences in physiology in pregnancy which necessitate the use of maternity specific severity scores. Patients who were discharged or died within the first 24 hours were also excluded.

Admissions with a blood culture were matched 1:1 to admissions without a blood culture who were in the same ward or clinical area at time of blood culture, (+/-24hrs for general wards and +/-96hrs for critical care), and who had similar length of hospital stay (+/-24hrs for general wards and +/-48hrs for critical care). Patients could be admitted more than once during the data collection period. Admissions which were more than 48hrs from previous discharge were included as the variable of interest was the coding applied in the study.

It was estimated from unpublished local data that 40–60% of patients with a blood culture taken during a hospital admission had sepsis. The sampling frame deliberately over-sampled patients likely to have infection as the aim of the case note review was to evaluate candidate sepsis detection and outcome tools rather than to estimate the incidence of sepsis. A target sample size of 1000 patients was estimated to provide sufficient data and is larger than most studies involving manual chart review. From matched admissions, all eligible critical care (intensive care, high dependency or coronary care) admissions were included, and a random sample of ward patients was selected to complete the cohort, using a computer-generated list of random numbers.

### Case note data

Case note review and data extraction were completed by a registered nurse (MB) using scanned paper clinical records. Demographic data included age, gender, and socioeconomic status. Postcode was used to allocate Scottish Index of Multiple Deprivation (SIMD), a Scottish Government measure of socioeconomic deprivation split into quintiles, with 1 being least affluent and 5 most affluent [25].

Each entire admission was manually reviewed for evidence of infection and categorised as the patient having no infection, infection without sepsis, or sepsis. Infection was defined as a documented source of infection and administration of either oral or intravenous antibiotics. The onset of infection was defined as the date and time of first blood culture taken or first antibiotic dose, whichever was earliest. As we were interested in identifying acute infection and sepsis, pre-existing infections with oral antibiotics prescribed in the community, prior to admission and without any escalation in hospital were excluded from the ‘infection’ group. A Consultant Physician in Infectious Diseases (CM) made the final decision on queries relating to the presence or absence of infection. Any documentation of suspected or confirmed sepsis by the clinical team was recorded during the case note review.

Physiological data to allow calculation of all the included severity scores were collected at the point of highest acuity, defined as the highest NEWS score in the 48hrs before or after onset of infection. Sepsis was defined as per Sepsis-3 [1]: infection plus a SOFA score of ≥ 2 for patients in general wards, or infection plus a change in SOFA score (measured once per 24hrs) of ≥2 for patients in critical care, in the 2 days before or after onset of infection.

### Administrative data

Data on blood culture sampling and results for the cohort were extracted by microbiology staff. Results were reviewed and categorised as negative, positive, or contaminated by a Consultant Physician in Infectious Diseases (CM) (S1 Table).

ICD-10 discharge codes assigned for each admission were extracted from Trakcare®, the hospital administration system. A list of infection and sepsis codes (S2 Table) was constructed, informed by the literature [24,26], by MB and CM.

The microbiology, ICD-10 and case note data were linked using a pseudo-anonymised patient CHI using a key, which was kept separately to the clinical data in a password-protected file on a secure server.

### Score calculation

Each patient with at least one episode of infection (as defined above) had the following scores calculated: SOFA, qSOFA, NEWS and SIRS (Table 1). NEWS and SIRS scores were calculated as per published tables [12,14]. As SOFA was designed for critical care it was anticipated that there would be a significant number of missing values for the respiratory component which requires an arterial blood gas to calculate the PaO_2_/FiO_2_ ratio. There are several available alternatives for ward patients [27,28] which use combinations of inspired oxygen and oxygen saturations. We used Valik et al’s [29] method which allocates a SOFA score of 1 for oxygen saturations of 91–94% and 2 for oxygen saturations of less than 91% as a replacement for the missing data because it performed better than either the original SOFA scoring [9] or Gadrey et al [27]. For both SOFA and qSOFA, AVPU (Alert, Verbal or Painful stimulus required to provoke a response, Unconscious) scoring was applied to calculate the neurological components when GCS was unavailable, as per the method published by Zadravecz et al. [30]. Other missing data were treated as normal values in score calculation.

**Table 1:** Severity score calculation: SOFA, qSOFA, NEWS & SIRS.

Severity Score	Scoring system
Sequential Organ Failure Assessment [9] (SOFA)	Sum of scores allocated for each physiological parameter^a^: PaO_2_/FiO_2_ or as per Valik [29], platelets, bilirubin, mean arterial pressure, Glasgow Coma Scale (or AVPU [30]) & creatinine/urine output
quick SOFA [1] (qSOFA)	One point for each of: Respiratory Rate ≥22 breaths/min, Systolic BP ≤100mmHg, Glasgow Coma Scale ≤13 (or AVPU <A [30])
National Early Warning Score [12] (NEWS)	Sum of scores allocated for each physiological parameter^b^: Respiratory rate, oxygen saturations, supplemental oxygen, temperature, systolic BP, heart rate, AVPU
Systemic Inflammatory Response Syndrome [14] (SIRS) Criteria	One point for each of: White Cell Count <4 x 10^9^ or >12 x10^9^, Heart Rate >90bpm, Respiratory Rate >20breaths/min, Temperature <36 or >38°C

^a^Each parameter allocated a score of 0–4, detailed in S3 Table.

^b^ Each parameter allocated a score of 0–3, detailed in S4 Table.

Calculation of NEWS, SIRS, qSOFA was undertaken using the physiological observations closest to time of onset of infection and also at the point of the highest NEWS in the 48hrs pre/post onset of infection. The highest NEWS score was identified and used in the analysis. SOFA was calculated using the worst blood results and worst physiological observations for each 24hr period in the 48hrs pre and post onset of infection. The highest SOFA score was used in the analysis.

Baseline SOFA score for patients in general wards was assumed to be zero, as per Sepsis-3^1^ guidance so a SOFA score of ≥2 related to infection was considered to be indicative of sepsis, whilst critical care patients required a *change* in SOFA score of ≥2, consequent to infection. A cut off score of ≥2 was applied to qSOFA [1] and SIRS [14]. Performance of NEWS was assessed using both the medium (NEWS ≥5) and high (NEWS ≥7) clinical risk thresholds [12].

### Statistical analysis

Descriptive statistics were used to summarise the cohort and compare groups. Pearson Chi-Square was calculated for categorical variables. Mann-Whitney U was applied to assess differences between continuous variables in two groups. Kruskall-Wallis was used to assess differences among more than two groups, on a single continuous variable. A *p* value of less than 0.05 was considered significant. No adjustment was made for multiplicity.

Performance characteristics (sensitivity, specificity, negative and positive predictive values, and receiver operating characteristic curves) of the severity scores were assessed for their ability to predict 30-day mortality from the onset of infection using the clinical cut-off scores for each tool. The area under the receiver operating characteristic curve (AUROC) and 95% confidence intervals were calculated for each severity score across all possible values of each score. Differences between AUROCs were assessed using DeLong’s test [31].

The performance characteristics and AUROC of administrative data items, singly and in combination, to identify people with sepsis were calculated, both for sepsis according to the Sepsis-3 SOFA definition, and to other severity score(s) performing best at predicting 30-day mortality after infection onset, i.e. that identified high risk infection patients.

Analyses were undertaken in SPSS version 25.0 (Armonk, NY, USA), and SAS version 9.4 (Cary, NC, USA).

## Results

**Fig 1:**
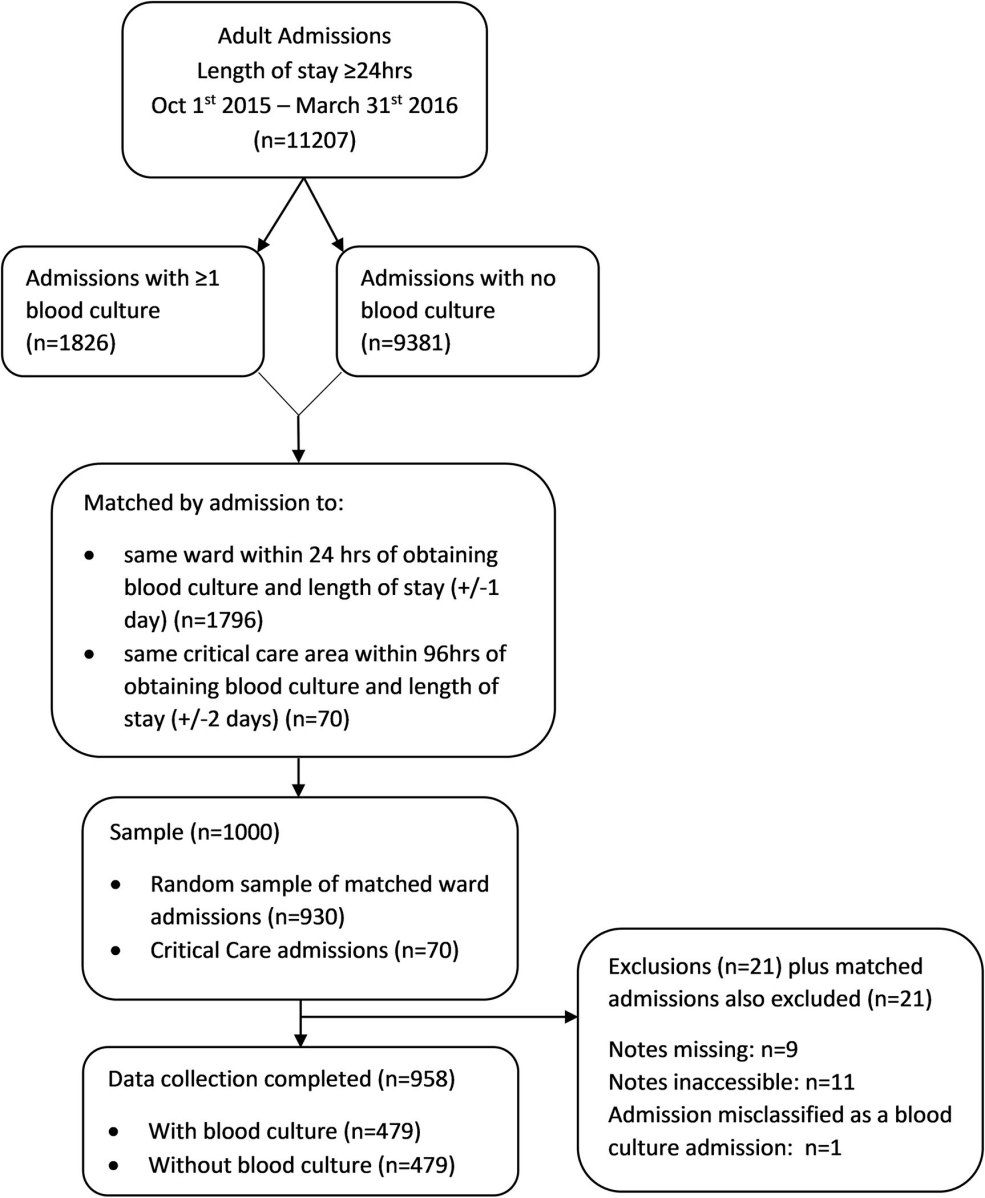
Cohort selection flow diagram.

During the study period there were 11207 adult admissions with a hospital length of stay of ≥24hrs (Fig 1). Of these, 9381 admissions had no blood culture and 1826 had at least one blood culture sample taken. Matching of admissions with and without a blood culture obtained 1796 matched ward admissions (898 matched pairs) and 70 matched critical care admissions (35 matched pairs).

All 70 critical care admissions were included and a simple random sample of 930 ward admissions (465 matched pairs) was obtained. Ultimately, 21 admissions were excluded due to inaccessible or missing clinical notes and matched admissions were also excluded, resulting in a final sample of 958 admissions.

### Study population

The final cohort (n = 958) had a median age of 68 (IQR 52–79) and 54% were female. The majority were emergency admissions (94.3%) and the cohort included both community-acquired infection and infection acquired during the hospital stay. Of the total cohort, 630 (65.7%) were treated for infection and of those, 347 (55.1%) had sepsis, defined using SOFA (Table 2). People with sepsis were older (*p* = <0.001), had a longer hospital stay (*p*<0.001) and were more likely to die within 30 days (*p*<0.001) compared to those without sepsis (Table 2). Only 32.6% (n = 113) of patients with sepsis, had suspected or confirmed sepsis documented contemporaneously in their medical notes.

**Table 2:** Demographics and clinical characteristics.

		All Patients (n = 958)	Sepsis^a^ (n = 347)	Infection without sepsis (n = 283)	No infection (n = 328)
Age, Median (IQR)		68 (52–79)	73 (59–82)	66 (47–77)	66 (50–77)
Female, n (%)		517 (54%)	171 (49.3%)	168 (59.4%)	178 (54.3%)
Scottish Index of Multiple Deprivation^b^	1 (least affluent)	342 (35.7%)	124 (35.7%)	100 (35.3%)	118 (36.0%)
	2	175 (18.3%)	71 (20.5%)	43 (15.2%)	61 (18.6%)
	3	195 (20.4%)	68 (19.6%)	61 (21.6%)	66 (20.1%)
	4	118 (12.3%)	44 (12.7%)	37 (13.1%)	37 (11.3%)
	5 (most affluent)	107 (11.2%)	32 (9.2%)	33 (11.7%)	42 (12.8%)
Emergency Admission, n (%)		903 (94.3%)	336 (96.8%)	276 (97.5%)	291 (88.7%)
Median Length of Stay, days (IQR)		4.5 (2.6–7.7)	5.8 (3.5–9.8)	3.9 (2.4–7.1)	3.8 (2.2–6.5)
Critical Care Admission, n (%)		92 (9.6%)	53 (15.3%)	17 (6.0%)	22 (6.7%)
Critical Care Admission 48hrs pre/post Infection, n (%)		59 (6.2%)	46 (13.3%)	13 (4.6%)	-
Blood culture obtained, n (%)		479 (50%)	229 (66.0%)	199 (70.3%)	51 (15.5%)
Positive blood culture, n (%)		42 (4.4%)	26 (7.5%)	16 (5.7%)	0 (0.0%)
In-hospital mortality, n (%)		72 (7.5%)	56 (16.1%)	7 (2.5%)	9 (2.7%)
30 Day Mortality from admission, n (%)		97 (10.1%)	69 (19.9%)	15 (5.3%)	13 (4.0%)
30 Day Mortality from onset of infection, n (%)		-	65 (18.7%)	13 (4.6%)	-

^a^According to Sepsis-3^1^ definition of sepsis as SOFA≥2, or change of ≥2 for critical care patients.

^b^ Patients with missing SIMD (n = 21) evenly distributed across groups.

In addition to the 62 admissions matched by critical care area at time of blood culture, 30 patients matched in general wards were admitted to critical care at some point during their stay. Of the 92 critical care patients, 59 (64.1%) were admitted to critical care within 48hrs pre or post onset of infection.

### Performance of SOFA, qSOFA, NEWS and SIRS in predicting mortality

Of the 630 patients with infection 55.1% had a SOFA score of ≥2 (or change of ≥2 for critical care patients), meeting the Sepsis-3^1^ criteria for sepsis (Table 3). 14.0% patients with infection had a qSOFA score of ≥2. Initial missing data for the respiratory component of SOFA (86.7%, n = 546) and the GCS component of qSOFA and SOFA (74.1%, n = 467) were replaced as described in Methods [29,30]. All other variables had little missing data (at least 93.5% completeness) with missing values assumed to be normal for the purpose of severity score calculation.

**Table 3:** Severity scores and performance characteristics for 30-day mortality from onset of infection.

	Patients with infection (n = 630)	30-day mortality on or above score cut off (%)	30-day mortality below cut off (%)	Performance characteristics for 30-day mortality			
				Sensitivity (95% CI)	Specificity (95% CI)	PPV* (95% CI)	NPV* (95% CI)
SOFA ≥2	347 (55.1%)	65/347 (18.7%)	13/283 (4.6%)	83.3% (73.2–90.8)	48.9% (44.7–53.2)	18.7% (14.8–23.2)	95.4% (92.3–97.5)
NEWS ≥5	336 (53.3%)	66/336 (19.6%)	12/294 (4.1%)	84.6% (74.7–91.8)	51.1% (46.8–55.3)	19.6% (15.5–24.3)	95.9% (93.0–97.9)
NEWS ≥7	200 (31.7%)	53/200 (26.5%)	25/430 (5.8%)	67.9% (56.4–78.1)	73.4% (69.5–77.0)	26.5% (20.5–33.2)	94.2% (91.5–96.2)
SIRS ≥2	405 (64.3%)	70/405 (17.3%)	8/225 (3.5%)	89.7% (80.8–95.5)	39.3% (35.2–43.5)	17.3% (13.7–21.3)	96.4% (93.1–98.5)
qSOFA ≥2	88 (14.0%)	33/88 (37.5%)	45/542 (8.3%)	42.3% (31.2–54.0)	90.0% (87.2–92.4)	37.5% (27.4–48.5)	91.7% (89.0–93.9)

*PPV: Positive Predictive Value; NPV: Negative Predictive Value; AUROC values calculated using cut-off points stated for each severity score.

The performance characteristics of each severity score in predicting 30-day mortality from onset of infection were calculated using the usual clinical cut-off scores (Table 3). A sensitivity analysis for in-hospital mortality is available as supplementary information (S5 Table). For predicting 30-day mortality qSOFA had the lowest sensitivity (42.3%%, 95%CI 31.2–54.0), but the highest specificity (90.0%, 95%CI 87.2–92.4%). SIRS had the highest sensitivity (89.7%, 95%CI 80.8–95.5%) but lowest specificity (39.3%, 95%CI 35.2–43.5%).

**Fig 2:**
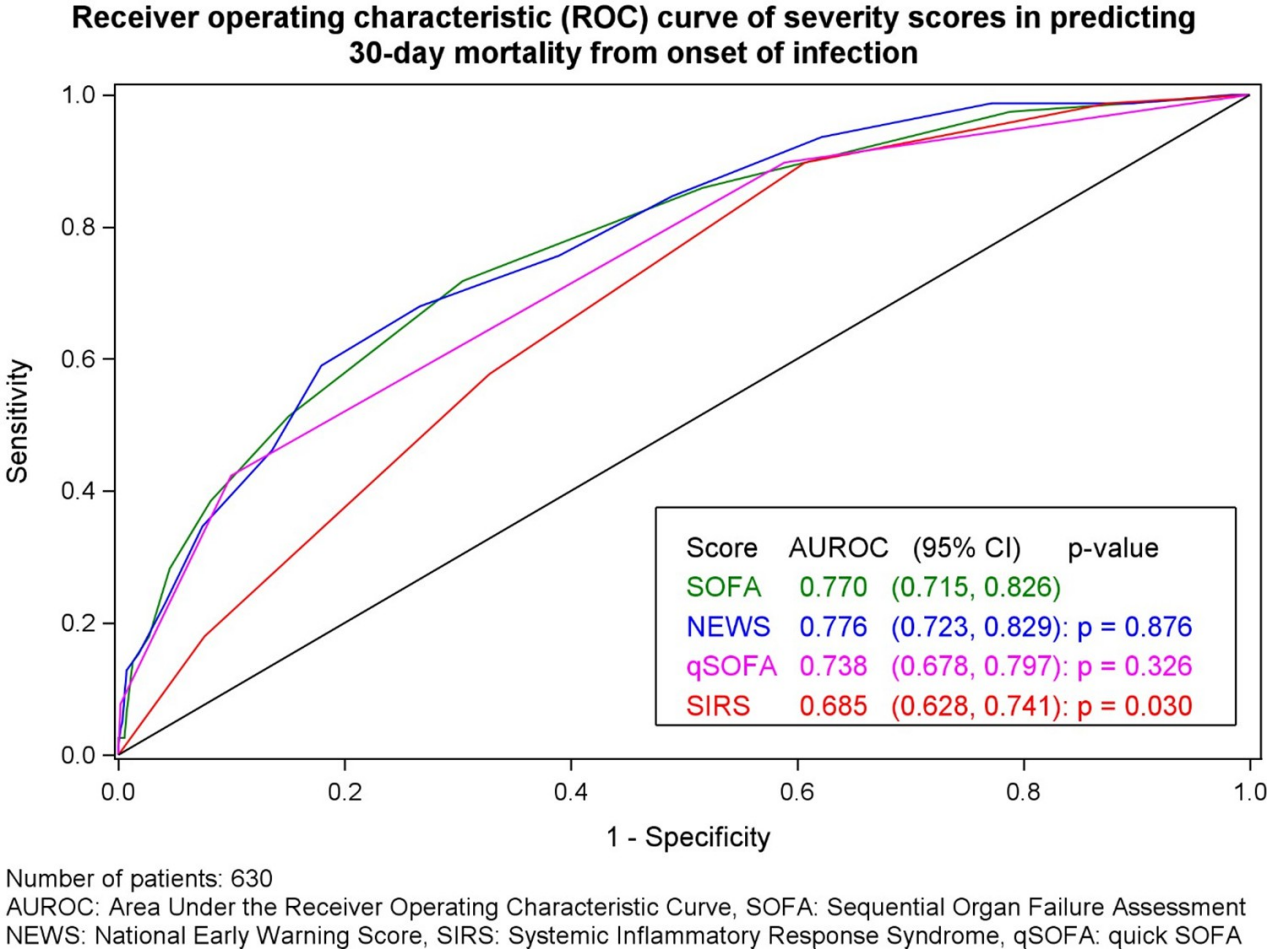
Receiver operating characteristic curves for 30-day mortality per severity score.

NEWS (AUROC 0.78, 95%CI 0.72–0.83*)* and SOFA (AUROC 0.77, 95%CI 0.72–0.83) performed similarly well for prediction of 30-day mortality from onset of infection when plotted across all values (Fig 2). qSOFA performed less well (AUROC 0.74, 95%CI 0.68–0.79) but the difference from SOFA was not statistically significant (DeLong’s test p = 0.33). SIRS had the poorest AUROC (0.68, 95%CI 0.63–0.74, DeLong’s test *p* = 0.03) (Fig 2).

The performance characteristics of each tool were also calculated separately for ward patients only to investigate the performance of SOFA in this cohort. For ward patients (n = 571) who were not admitted to critical care during the 48hrs pre or post onset of infection NEWS predicted 30-day mortality (AUROC 0.80, 95%CI 0.73–0.86) better across all values than any other tool (SOFA, 0.73, 95%CI 0.66–0.80; SIRS, 0.70, 95% CI 0.64–0.77; qSOFA, 0.72, 95%CI 0.65–0.80), although the difference for SOFA was not quite statistically significant (DeLong’s p = 0.07), in predicting 30-day mortality but performed significantly better than SIRS (p = 0.003) or qSOFA (p = 0.007).

### Performance characteristics of routine data to identify people with sepsis

Only 27 (7.8%) sepsis admissions had a sepsis ICD-10 code (A40, A41 or R57.2) (Table 4). Coding for infection was more frequent with 226 (65.1%) of sepsis admissions being allocated at least one infection code. More than 80% of infection and sepsis codes allocated were from three ICD-10 chapters: diseases of the respiratory system (J00-J99), certain infectious and parasitic diseases (A00-B99) and diseases of the genitourinary system (N00-N99).

**Table 4:** Performance characteristics of administrative data to identify people with sepsis.

	Sepsis			Sensitivity (95% CI)	Specificity (95% CI)	PPV* (95% CI)	NPV* (95% CI)	AUROC (95% CI)
		Yes	No					
Infection code	Yes	226	198	65.1% (59.9–70.1)	67.6% (63.7–71.3)	53.3% (48.4–58.1)	77.3% (73.6–80.8)	0.66 (0.63–0.70)
	No	121	413					
Sepsis code	Yes	27	15	7.8% (5.2–11.1)	97.5% (96.0–98.6)	64.3% (48.0–78.4)	65.1% (61.9–68.2)	0.53 (0.49–0.57)
	No	320	596					
Infection or sepsis code	Yes	243	210	70.0% (64.9–74.8)	65.6% (61.7–69.4)	53.6% (48.9–58.3)	79.4% (75.6–82.9)	0.68 (0.64–0.71)
	No	104	401					
Blood culture	Yes	229	250	66.0% (60.7–71.0)	59.1% (55.1–63.0)	47.8% (43.3–52.4)	75.4% (71.3–79.2)	0.63 (0.59–0.66)
	No	118	361					
Positive blood culture	Yes	26	16	7.5% (5.0–10.8)	97.4% (95.8–98.5)	61.9% (45.6–76.4)	65.0% (61.8–68.0)	0.52 (0.49–0.56)
	No	321	595					
Infection or sepsis code or blood culture	Yes	303	313	87.3% (83.4–90.6)	48.8% (44.7–52.8)	49.2% (45.2–53.2)	87.1% (83.1–90.5)	0.68 (0.65–0.71)
	No	44	298					
Infection or sepsis code plus blood culture	Yes	169	147	48.7% (43.3–54.1)	75.9% (72.3–79.3)	53.5% (47.8–59.1)	72.3% (68.6–75.7)	0.62 (0.59–0.66)
	No	178	464					

*PPV: Positive Predictive Value; NPV: Negative Predictive Value.

Having a blood culture taken (AUROC 0.63, 95%CI 0.59–0.66) and infection codes (AUROC 0.66, 95%CI 0.63–0.70) had higher AUROCs to identify people with sepsis, defined by Sepsis-3 SOFA critiera, than either a positive blood culture result (AUROC 0.52, 95%CI 0.49–0.56), or a sepsis ICD-10 code (A40, A41, R57.2) (AUROC 0.53, 95%CI 0.49–0.57) (Table 4). In combination, having an infection code and/or a sepsis code (AUROC 0.68, 95%CI 0.64–0.71) performed the same as having at least one of: an infection code; a sepsis code, or; a blood culture (AUROC 0.68, 95%CI 0.65–0.71).

The performance of administrative data in identifying people with infection and NEWS ≥7 (as an alternative sepsis identification method) was similar to its ability in identifying people meeting the Sepsis-3 SOFA criteria (S6 Table).

## Discussion

To the best of our knowledge this is the first study which investigates the performance of qSOFA, SIRS, NEWS and SOFA in predicting 30-day mortality of patients with infection in a general hospital cohort. For sepsis outcome surveillance using routinely collected administrative data, ICD-10 sepsis codes and positive blood cultures were insufficiently sensitive but blood culture sampling and infection codes both performed better.

Screening patients for infection across the whole hospital stay rather than only at admission was a strength of this study and provided a complete picture of sepsis incidence within the cohort. The use of clinical data to identify people with sepsis also avoided the bias of selecting patients on the basis of their ICD-10 codes and enabled assessment of coding patterns. We were also able to detect mortality following discharge.

This study was limited by its retrospective, single centre design. Although excluding patients who died or were discharged within 24 hours of admission could have introduced selection bias, they were unlikely to have sufficient physiological data for the purposes of this study, which may have skewed the performance characteristics of the severity scores and administrative data. The need to replace AVPU and the respiratory component of SOFA due to the lack of recorded GCS, used in qSOFA and SOFA, and arterial blood gas values, used in SOFA, was unsurprising given that general ward patients often do not require arterial blood gas sampling and would not routinely have a GCS recorded unless there was concern about their neurological status. Other studies have also noted GCS as often not recorded [32].

Whilst assuming a baseline SOFA score of zero in ward patients is recommended in the Sepsis-3 guidelines [1], it is acknowledged that the lack of a true pre-admission SOFA score has potential to bias the results [27].

Our findings are consistent with some of the broader literature demonstrating superior performance of NEWS over qSOFA and SIRS in predicting mortality, [15,33–35] although other studies have reported SOFA as being superior to NEWS in predicting mortality [16,36]. SOFA was, however, designed and largely studied for use within critical care settings and its prognostic accuracy has been less thoroughly evaluated within the general ward population. Our findings add to the body of knowledge on SOFA performance outside of critical care.

The suboptimal performance of qSOFA in predicting mortality, particularly in terms of sensitivity, is similar to that reported elsewhere [37–39]. Our study also found that, compared to NEWS, qSOFA did not perform well in identifying people who met the Sepsis-3 SOFA criteria (infection plus SOFA ≥2 or change of 2 for critical care admissions). These findings add to the evidence challenging the use of qSOFA over NEWS [11,15]. Similar to other studies, SIRS had a high sensitivity but poor specificity and as such is a poor predictor of those patients with infection at an increased risk of a poor outcome [15,34].

In line with other studies [29,40], our research reported significant under coding for sepsis and consequent poor performance of the ICD-10 sepsis codes in identifying patients with sepsis. The growth in sepsis coding observed in the literature has contributed to reporting of significant increases in documented sepsis incidence [26,41,42]. Our finding of lack of sensitivity in sepsis codes and lack of specificity in infection codes, supports concerns about the quality of coding, its impact on epidemiological trends and potential for misinformed influence on policy and practice [17,42].

There is a growing body of literature which demonstrates the potential of machine learning in predicting sepsis in individual patients [43,44] and therefore also theoretical future capacity for sepsis surveillance at scale. This however requires fully automated electronic health records which are not yet standard in the UK or other countries in the Global North. Consequently, there remains a need for an objective, stable and clinically relevant proxy measure for sepsis surveillance. Positive blood cultures predictably performed poorly as a surrogate marker for sepsis in our cohort and, as reported elsewhere, lacks the sensitivity required for sepsis surveillance [45]. Blood culture sampling alone, however, performed significantly better than ICD-10 sepsis codes in identifying patients with sepsis, demonstrating potential as a component of routine sepsis surveillance. As an administrative data variable, blood culture sampling is unaffected by trends in diagnostic coding but is susceptible to changes and local variation in sampling practice. Further study is required to assess the effect that standardisation of blood culture sampling in sepsis has on its sensitivity and specificity as a measure.

Although no one combination of administrative data variables had an AUROC of >0.7, generally considered to be the cut-off for acceptable discrimination [46], this study did identify blood cultures as data routinely collected in Scotland with better predictive accuracy for sepsis than the sepsis explicit ICD-10 codes currently used. Blood culture data could offer an objective adjunct to coding with potential for application at a population level in contexts where it is electronically available at scale. The development of a stable measure to identify people with sepsis from administrative data would make a significant contribution to both sepsis surveillance and, more broadly, to the decisions policy makers make in relation to resource allocation in a fiscally pressured system. It would also address the concerns raised about potential for instability in coding and over-diagnosis [26,42]. Further investigation and validation of a combined measure is required in a larger, multicentre cohort.

## Conclusions

Our results support the use of SOFA and NEWS ≥7 to identify patients with infection at increased risk of death at 30 days, from onset of infection. The use of sepsis explicit ICD-10 codes to identify people with sepsis performed poorly in this cohort. Blood cultures as clinical, but routinely stored data, demonstrated potential as one component of a surrogate marker for sepsis, along with infection ICD-10 codes. This study provides important insights into potential use of administrative data for sepsis surveillance at scale.

## Supporting information

S1 Table Organisms grown from blood cultures sampled from study cohort. (DOCX)

S2 Table ICD-10 infection and sepsis codes reported in this cohort. (DOCX)

S3 Table Sequential Organ Failure Assessment (SOFA) scoring system. Vincent JL, Moreno R, Takala J. Willatts S, De Mendonҫa A, Bruining H, et al. Working Group on Sepsis-Related Problems of the European Society of Intensive Care Medicine. The SOFA (Sepsis-related Organ Failure Assessment) score to describe organ dysfunction/failure. Intensive Care Medicine. 1996; 22(7):707–710.
(DOCX)

S4 Table National Early Warning Score (NEWS). Royal College of Physicians. National Early Warning Score (NEWS). Standardising the assessment of acute-illness severity in the NHS. 2012. London: Royal College of Physicians.
(DOCX)

S5 Table Performance characteristics of severity scores to predict in-hospital mortality. (DOCX)

S6 Table Performance characteristics of administrative data to identify patients with infection & NEWS ≥7. *PPV: Positive Predictive Value; NPV: Negative Predictive Value.
(DOCX)

S1 Dataset Minimum dataset for the study. (XLSX)
