# Supplementary material for: Performance of bedside tools for predicting infection-related mortality and administrative data for sepsis surveillance: An observational cohort study
Source: PLoS One. 2023 Mar 2;18(3):e0280228. doi: 10.1371/journal.pone.0280228 (PMC9980760; doi:10.1371/journal.pone.0280228)
Supplement: S1 Table — (DOCX) [file pone.0280228.s001.docx]

S1 Table. Organisms grown from blood cultures sampled from study cohort.

| **Organism** | **Positive** | **Contaminant** |
| --- | --- | --- |
| Actinomyces odontolyticus | 1 | 0 |
| Alpha Haemolytic Strep | 1 | 0 |
| Clostridium sordellii | 1 | 0 |
| Coagulase-Negative Staphylococcus | 0 | 1 |
| Enterococcus faecalis, | 1 | 0 |
| Enterococcus faecium | 1 | 0 |
| Escherichia coli | 1 | 0 |
| Group B Streptococcus | 1 | 0 |
| Klebsiella oxytoca | 1 | 0 |
| Klebsiella pneumoniae | 1 | 0 |
| Leuconostoc mesenteroides | 0 | 1 |
| Micrococcus luteus/lylae | 0 | 1 |
| Neisseria meningitidis | 1 | 0 |
| Propionibacterium acnes | 0 | 1 |
| Propionibacterium species | 0 | 1 |
| Proteus mirabilis | 1 | 0 |
| Staphylococcus aureus | 1 | 0 |
| Staphylococcus capitis | 0 | 1 |
| Staphylococcus epidermidis | 0 | 1 |
| Staphylococcus haemolyticus, | 0 | 1 |
| Staphylococcus hominis ssp hominis | 0 | 1 |
| Staphylococcus warneri | 0 | 1 |
| Streptococcus mitis/Streptococcus oralis | 1 | 0 |
| Streptococcus gordonii, | 1 | 0 |
| Streptococcus parasanguinis | 1 | 0 |
| Streptococcus pneumoniae | 1 | 0 |
| Streptococcus salivarius | 1 | 0 |
