# Supplementary material for: Performance of bedside tools for predicting infection-related mortality and administrative data for sepsis surveillance: An observational cohort study
Source: PLoS One. 2023 Mar 2;18(3):e0280228. doi: 10.1371/journal.pone.0280228 (PMC9980760; doi:10.1371/journal.pone.0280228)
Supplement: S2 Table — (DOCX) [file pone.0280228.s002.docx]

The following ICD-10 infection and sepsis codes were reported in this cohort.

| **ICD-10 Code** | **ICD10 code Descriptor** |
| --- | --- |
| A02.9 | SALMONELLA INFECTION, UNSPECIFIED |
| A04.4 | OTHER INTESTINAL ESCHERICHIA COLI INFECTIONS |
| A04.5 | CAMPYLOBACTER ENTERITIS |
| A04.7 | ENTEROCOLITIS DUE TO CLOSTRIDIUM DIFFICILE |
| A15.0 | T.B. OF LUNG, CONFIRMED BY SPUTUM MICROSCOPY WITH OR WITHOUT CULTURE |
| A40.1 | SEPSIS DUE TO STREPTOCOCCUS, GROUP B |
| A40.8 | OTHER STREPTOCOCCAL SEPSIS |
| A41.2 | SEPSIS DUE TO UNSPECIFIED STAPHYLOCOCCUS |
| A41.5 | SEPSIS DUE TO OTHER GRAM-NEGATIVE ORGANISMS |
| A41.9 | SEPSIS, UNSPECIFIED |
| A48.0 | GAS GANGRENE |
| A49.0 | STAPHYLOCOCCAL INFECTION, UNSPECIFIED SITE |
| A49.1 | STREPTOCOCCAL AND ENTEROCOCCAL INFECTION, UNSPECIFIED SITE |
| A49.8 | OTHER BACTERIAL INFECTIONS OF UNSPECIFIED SITE |
| B95.6 | STAPH AUREUS AS THE CAUSE OF DISEASES CLASSIFIED TO OTHER CHAPTERS |
| B96.1 | K. PNEUMONIAE AS THE CAUSE OF DISEASES CLASSIFIED TO OTHER CHAPTERS |
| B96.2 | E. COLI AS THE CAUSE OF DISEASES CLASSIFIED TO OTHER CHAPTERS |
| B96.3 | H. INFLUENZAE AS THE CAUSE OF DISEASES CLASSIFIED TO OTHER CHAPTERS |
| B96.5 | PSEUDOMONAS (AERUGINOSA) AS THE CAUSE OF OTHER DISEASES |
| B96.6 | B. FRAGILIS AS THE CAUSE OF DISEASES CLASSIFIED TO OTHER CHAPTERS |
| B96.7 | C. PERFRINGENS AS THE CAUSE OF DISEASES CLASSIFIED TO OTHER CHAPTERS |
| B96.8 | OTHER SPEC BACTERIA AS THE CAUSE OF DISEASES CLASSIFIED TO OTHER CHAPTERS |
| H66.9 | OTITIS MEDIA, UNSPECIFIED |
| J02.9 | ACUTE PHARYNGITIS, UNSPECIFIED |
| J03.9 | ACUTE TONSILLITIS, UNSPECIFIED |
| J06.9 | ACUTE UPPER RESPIRATORY INFECTION, UNSPECIFIED |
| J13.X | PNEUMONIA DUE TO STREPTOCOCCUS PNEUMONIAE |
| J15.9 | BACTERIAL PNEUMONIA, UNSPECIFIED |
| J18.0 | BRONCHOPNEUMONIA, UNSPECIFIED |
| J18.1 | LOBAR PNEUMONIA, UNSPECIFIED |
| J18.9 | PNEUMONIA, UNSPECIFIED |
| J22.X | UNSPECIFIED ACUTE LOWER RESPIRATORY INFECTION |
| J44.0 | CHRONIC OBSTRUCTIVE PULMONARY DISEASE WITH ACUTE LOWER RESP INFECTION |
| J69.0 | PNEUMONITIS DUE TO FOOD AND VOMIT |
| J85.2 | ABSCESS OF LUNG WITHOUT PNEUMONIA |
| J86.9 | PYOTHORAX WITHOUT FISTULA |
| K35.2 | ACUTE APPENDICITIS WITH GENERALIZED PERITONITIS |
| **ICD-10 Code** | **ICD10 code Descriptor** |
| K35.3 | ACUTE APPENDICITIS WITH LOCALIZED PERITONITIS |
| K35.8 | ACUTE APPENDICITIS, OTHER AND UNSPECIFIED |
| K57.2 | DIVERTICULAR DISEASE OF LARGE INTESTINE WITH PERFORATION AND ABSCESS |
| K57.8 | DIVERTICULAR DISEASE OF INTESTINE, PART UNSPECIFIED, WITH PERF AND ABSCESS |
| K61.0 | ANAL ABSCESS |
| K61.3 | ISCHIORECTAL ABSCESS |
| K63.0 | ABSCESS OF INTESTINE |
| K63.1 | PERFORATION OF INTESTINE (NONTRAUMATIC) |
| K65.9 | PERITONITIS, UNSPECIFIED |
| K75.0 | ABSCESS OF LIVER |
| K80.0 | CALCULUS OF GALLBLADDER WITH ACUTE CHOLECYSTITIS |
| K80.1 | CALCULUS OF GALLBLADDER WITH OTHER CHOLECYSTITIS |
| K80.3 | CALCULUS OF BILE DUCT WITH CHOLANGITIS |
| K80.4 | CALCULUS OF BILE DUCT WITH CHOLECYSTITIS |
| K81.0 | ACUTE CHOLECYSTITIS |
| K81.9 | CHOLECYSTITIS, UNSPECIFIED |
| K83.0 | CHOLANGITIS |
| L02.2 | CUTANEOUS ABSCESS, FURUNCLE AND CARBUNCLE OF TRUNK |
| L02.3 | CUTANEOUS ABSCESS, FURUNCLE AND CARBUNCLE OF BUTTOCK |
| L03.1 | CELLULITIS OF OTHER PARTS OF LIMB |
| L03.2 | CELLULITIS OF FACE |
| L03.3 | CELLULITIS OF TRUNK |
| L03.9 | CELLULITIS, UNSPECIFIED |
| L05.0 | PILONIDAL CYST WITH ABSCESS |
| L08.9 | LOCAL INFECTION OF SKIN AND SUBCUTANEOUS TISSUE, UNSPECIFIED |
| M00.96 | PYOGENIC ARTHRITIS, UNSPECIFIED (LOWER LEG) |
| M71.15 | OTHER INFECTIVE BURSITIS (PELVIC/THIGH) |
| M86.17 | OTHER ACUTE OSTEOMYELITIS (ANKLE/FOOT) |
| M86.69 | OTHER CHRONIC OSTEOMYELITIS (SITE UNSPEC) |
| N10.X | ACUTE TUBULO-INTERSTITIAL NEPHRITIS |
| N11.9 | CHRONIC TUBULO-INTERSTITIAL NEPHRITIS, UNSPECIFIED |
| N12.X | TUBULO-INTERSTITIAL NEPHRITIS, NOT SPECIFIED AS ACUTE OR CHRONIC |
| N13.6 | PYONEPHROSIS |
| N39.0 | URINARY TRACT INFECTION, SITE NOT SPECIFIED |
| N45.0 | ORCHITIS, EPIDIDYMITIS AND EPIDIDYMO-ORCHITIS WITH ABSCESS |
| N45.9 | ORCHITIS, EPIDIDYMITIS AND EPIDIDYMO-ORCHITIS WITHOUT ABSCESS |
| R02.X | GANGRENE, NOT ELSEWHERE CLASSIFIED |
| R57.2 | SEPTIC SHOCK |
| T80.2 | INFECTIONS FOLLOWING INFUSION, TRANSFUSION AND THERAPEUTIC INJECTION |
| T81.4 | INFECTION FOLLOWING A PROCEDURE NOT ELSEWHERE CLASSIFIED |
| T83.5 | INF/INFLAMM REACTION DUE TO PROSTH DEVICE/IMPLANT/GRAFT IN URINARY SYST |
| T84.5 | INFECTION AND INFLAMMATORY REACTION DUE TO INTERNAL JOINT PROSTHESIS |
| T857 | INFECTION AND INFLAMMATORY REACTION DUE TO OTH INTERNAL PROSTHETIC DEVICES IMPLANTS AND GRAFTS |
