# Supplementary material for: Performance of bedside tools for predicting infection-related mortality and administrative data for sepsis surveillance: An observational cohort study
Source: PLoS One. 2023 Mar 2;18(3):e0280228. doi: 10.1371/journal.pone.0280228 (PMC9980760; doi:10.1371/journal.pone.0280228)
Supplement: S3 Table — Vincent JL, Moreno R, Takala J. Willatts S, De Mendonҫa A, Bruining H, et al. Working Group on Sepsis-Related Problems of the European Society of Intensive Care Medicine. The SOFA (Sepsis-related Organ Failure Assessment) score to describe organ dysfunction/failure. Intensive Care Medicine. 1996; 22(7):707–710. (DOCX) [file pone.0280228.s003.docx]

**Table S3. Sequential Organ Failure Assessment (SOFA) scoring system**

| **Sequential Organ Failure Assessment (SOFA) ≥2** | | | | | |
| --- | --- | --- | --- | --- | --- |
| System | **Score** | | | | |
|  | 0 | 1 | 2 | 3 | 4 |
| **Respiratory**  PaO2/FiO2, kPa | ≥53.3 | <53.3 | <40 | <26.7 with respiratory support | <13.3 with respiratory support |
| **Coagulation**  Platelets, x10^3^/microliters | ≥150 | <150 | <100 | <50 | <20 |
| **Liver**  Bilirubin, µmol/L | <20 | 20-32 | 33-101 | 102-204 | >204 |
| **Cardiovascular** | MAP ≥70mmHg | MAP <70mmHg | Dopamine <5µg/kg/min or dobutamine (any dose) | Dopamine 5.1-15 or adrenaline ≤0.1 or noradrenaline ≤0.1 (all units µg/kg/min) | Dopamine >15 or adrenaline >0.1 or noradrenaline >0.1 (all units µg/kg/min) |
| **Central nervous system**  Glasgow Coma Scale | 15 | 13-14 | 10-12 | 6-9 | <6 |
| **Renal**  Creatinine, µmol/L  Urine output, ml/d | <110 | 110-170 | 171-299 | 300-440 | >440 |
|  |  |  |  | <500ml | <200ml |

Vincent JL, Moreno R, Takala J. Willatts S, De Mendonҫa A, Bruining H, Reinhart CK, Surter PM, Thijs LG. Working Group on Sepsis-Related Problems of the European Society of Intensive Care Medicine. The SOFA (Sepsis-related Organ Failure Assessment) score to describe organ dysfunction/failure. Intensive Care Medicine. 1996;22(7):707-710
