# Supplementary material for: Performance of bedside tools for predicting infection-related mortality and administrative data for sepsis surveillance: An observational cohort study
Source: PLoS One. 2023 Mar 2;18(3):e0280228. doi: 10.1371/journal.pone.0280228 (PMC9980760; doi:10.1371/journal.pone.0280228)
Supplement: S4 Table — Royal College of Physicians. National Early Warning Score (NEWS). Standardising the assessment of acute-illness severity in the NHS. 2012. London: Royal College of Physicians. (DOCX) [file pone.0280228.s004.docx]

**Table S4. National Early Warning Score (NEWS)**

| **National Early Warning Score (NEWS) of ≥5 or ≥7** | | | | | | | |
| --- | --- | --- | --- | --- | --- | --- | --- |
|  | **3** | **2** | **1** | **0** | **1** | **2** | **3** |
| Respiratory Rate (bpm) | ≤8 |  | 9-11 | 12-20 |  | 21-24 | ≥25 |
| Oxygen Saturations (%) | ≤91 | 92-93 | 94-95 | ≥96 |  |  |  |
| Supplemental oxygen |  | Yes |  | None |  |  |  |
| Temperature (°C) | ≤35.0 |  | 35.1-36.0 | 36.1-38.0 | 38.1-39.0 | ≥39.1 |  |
| Systolic BP | ≤90 | 91-100 | 101-110 | 111-219 |  |  | ≥220 |
| Heart Rate | ≤40 |  | 41-50 | 51-90 | 91-110 | 111-130 | ≥131 |
| Level of Consciousness (AVPU) |  |  |  | Alert |  |  | VPU |

Royal College of Physicians. National Early Warning Score (NEWS). Standardising the assessment of acute-illness severity in the NHS. 2012. London: Royal College of Physicians.
