# Supplementary material for: Performance of bedside tools for predicting infection-related mortality and administrative data for sepsis surveillance: An observational cohort study
Source: PLoS One. 2023 Mar 2;18(3):e0280228. doi: 10.1371/journal.pone.0280228 (PMC9980760; doi:10.1371/journal.pone.0280228)
Supplement: S5 Table — (DOCX) [file pone.0280228.s005.docx]

S5. Table. Severity Score Performance characteristics in predicting in-hospital mortality in patients with suspected or confirmed infection

|  | Patients with infection (n=630) | Died in hospital (n=63) | Performance characteristics for in-hospital mortality | | | |
| --- | --- | --- | --- | --- | --- | --- |
|  |  |  | Sensitivity (95% CI) | Specificity (95% CI) | PPV*  (95% CI) | NPV*  (95% CI) |
| SOFA ≥2^a^ | 347  (55.1%) | 56  (88.9%) | 88.9%  (78.4-95.4) | 48.7%  (44.5-52.9) | 16.1%  (12.4-20.4) | 97.5%  (95.0-99.0) |
| NEWS ≥5 | 336  (53.3%) | 55  (87.3%) | 87.3%  (76.5-94.4) | 50.4%  (46.2-54.6) | 16.4%  (12.6-20.8) | 97.3%  (94.7-98.8) |
| NEWS ≥7 | 200  (31.7%) | 45  (71.4%) | 71.4%  (58.7-82.1) | 72.7%  (68.8-76.3) | 22.5%  (16.9-28.9) | 95.8%  (93.5-97.5) |
| SIRS ≥2 | 405  (64.3%) | 56  (88.9%) | 88.9%  (78.4-95.4) | 38.4%  (34.4-42.6) | 13.8%  (10.6-17.6) | 96.9%  (93.7-98.7) |
| qSOFA ≥2^b^ | 88  (14.0%) | 30  (47.6%) | 47.6%  (34.9-60.6) | 89.8%  (87.0-92.1) | 34.1%  (24.3-45.0) | 93.9%  (91.6-95.8) |

^a^SOFA calculated using Valik method and including AVPU in place of missing GCS; ^b^qSOFA calculated using AVPU in place of missing GCS using ref ref method
