# Supplementary material for: Performance of bedside tools for predicting infection-related mortality and administrative data for sepsis surveillance: An observational cohort study
Source: PLoS One. 2023 Mar 2;18(3):e0280228. doi: 10.1371/journal.pone.0280228 (PMC9980760; doi:10.1371/journal.pone.0280228)
Supplement: S6 Table — *PPV: Positive Predictive Value; NPV: Negative Predictive Value. (DOCX) [file pone.0280228.s006.docx]

S6. Table. Administrative data performance in identifying patients with infection and NEWS≥7

|  | NEWS ≥7 | | | Sensitivity (95% CI) | Specificity (95% CI) | PPV*  (95% CI) | NPV*  (95% CI) | AUROC (95% CI) |
| --- | --- | --- | --- | --- | --- | --- | --- | --- |
|  |  | Yes | No |  |  |  |  |  |
| Infection code | Yes | 133 | 291 | 66.5% (59.5-73.0) | 61.6% (58.0-65.1) | 31.4% (27.0-36.0) | 87.5% (84.3-90.1) | 0.64  (0.60-0.68) |
|  | No | 67 | 467 |  |  |  |  |  |
| Sepsis code | Yes | 12 | 30 | 6.0%  (3.1-10.2) | 96.0%  (94.4-97.3) | 28.6%  (15.7-44.6) | 79.5%  (76.7-82.0) | 0.51  (0.47-0.56) |
|  | No | 188 | 728 |  |  |  |  |  |
| Infection or sepsis code | Yes | 141 | 312 | 70.5% (63.7-76.7) | 58.8% (55.2-62.4) | 31.1% (26.9-35.6) | 88.3% (85.2-91.0) | 0.65  (0.61-0.69) |
|  | No | 59 | 446 |  |  |  |  |  |
| Blood culture taken | Yes | 154 | 325 | 77.0%  (70.5-82.6) | 57.1%  (53.5-60.7) | 32.2%  (28.0-36.5) | 90.4%  (87.4-92.9) | 0.67  (0.63-0.71) |
|  | No | 46 | 433 |  |  |  |  |  |
| Positive blood culture result | Yes | 15 | 27 | 7.5%  (4.3-12.1) | 96.4%  (94.9-97.6) | 35.7%  (21.6-52.0) | 79.8%  (77.1-82.4) | 0.52  (0.47-0.57) |
|  | No | 185 | 731 |  |  |  |  |  |
| Infection or sepsis code or blood culture taken | Yes | 184 | 432 | 92.0% (87.3-95.4) | 43.0% (39.5-46.6) | 29.9% (26.3-33.7) | 95.3% (92.5-97.3) | 0.68 (0.64-0.71) |
|  | No | 16 | 326 |  |  |  |  |  |
| Infection or sepsis code plus blood culture taken | Yes | 111 | 205 | 55.5% (48.3-62.5) | 73.0% (69.6-76.1) | 35.1% (29.9-40.7) | 86.1% (83.2-88.7) | 0.64  (0.60-0.69) |
|  | No | 89 | 553 |  |  |  |  |  |

*PPV: Positive Predictive Value; NPV: Negative Predictive Value.
